# Interactive effect of *MTHFR* C677T polymorphism and sex on symptoms and cognitive functions in Chinese patients with chronic schizophrenia

**DOI:** 10.18632/aging.103248

**Published:** 2020-06-04

**Authors:** Jie Gao, Mei Hong Xiu, Dian Ying Liu, Chang Wei Wei, Xiangyang Zhang

**Affiliations:** 1 Department of Cardiac Surgery, Beijing Chao-yang Hospital, Capital Medical University, Beijing, China; 2 Beijing HuiLongGuan Hospital, Peking University HuiLongGuan Clinical Medical School, Beijing, China; 3 Department of Psychiatry, The Third People’s Hospital of Ganzhou, Ganzhou, Jiangxi, China; 4 Department of Anesthesiology, Beijing Chao-Yang Hospital, Capital Medical University, Beijing, China; 5 CAS Key Laboratory of Mental Health, Institute of Psychology, Chinese Academy of Sciences, Beijing, China

**Keywords:** schizophrenia, symptoms, cognition, MTHFR, polymorphism

## Abstract

The etiology of schizophrenia is still unknown, and the *MTHFR* gene has been shown to be associated with SCZ. Previous studies have shown that patients with schizophrenia exhibit sex differences in symptoms and cognitive function. However, no study has been conducted to investigate the sex difference in the association between C677T polymorphism and symptoms and cognitive impairment in Chinese patients with schizophrenia. The C677T polymorphism was genotyped in 957 patients with schizophrenia and 576 controls. Patients were also rated on the Positive and Negative Syndrome Scale (PANSS) and the Repeatable Battery for the Assessment of Neuropsychological Status (RBANS). The results showed that there were significant differences in *MTHFR* C677T genotype and allele distributions between male patients and male controls (both p<0.05), while there was no significant difference between female patients and female controls (both p>0.05). Further analysis showed that there were significant sex differences in the association between C677T genotype and negative symptoms, immediate memory or attention index score in schizophrenia (p<0.05). This study suggests that the complex interactive effect between *MTHFR* C677T polymorphism and sex plays an important role in some clinical characteristics of patients with schizophrenia.

## INTRODUCTION

Patients with schizophrenia (SCZ) exhibit cognitive impairments in several domains throughout the disease process [1, 2]. Cognitive impairment is recognized to be one of the core characteristics of SCZ, which includes attention, working memory, verbal learning and memory, and executive function [3]. However, the pathophysiological mechanisms of cognitive impairment in SCZ patients are still unclear, especially the biological pathological mechanism.

Accumulating studies have revealed that there are sex differences in the features of SCZ from the prevalence, symptoms, age at onset, illness course, to response to treatment [4–7]. For example, several reviews have demonstrated that female patients have an advantage over male patients in terms of age of onset, response to antipsychotic treatment, social function, and clinical symptoms, especially negative symptoms [4, 8–10]. However, female patients have more severe lipid metabolic dysfunction and more obesity than male patients [11]. More interestingly, some studies have investigated sex differences in cognitive impairment in patients with SCZ [4, 12, 13], indicating that men performed worse in attention, language and verbal memory and executive function [12, 14]. However, the results of sex differences in cognitive impairments and clinical symptoms were contradictory [15, 16]. Several studies have reported that there is no sex difference in cognitive impairment in patients with SCZ or even opposite results [16].

The methylenetetrahydrofolate reductase *(MTHFR)* gene is located on chromosome 1 and contains several common polymorphisms in its exons. The substitution (C to T) of nucleotide 677 in exon 4 results in amino acid substitution (Ala222Val) and decreased MTHFR activity [17]. Decreased MTHFR metabolism leads to hypomethylation of DNA, increases the concentrations of potentially toxic homocysteine, and causes abnormal levels of neurotransmitters [18–20].

Recently, the *MTHFR* C677T polymorphism has consistently reported to be related to SCZ, which has also been confirmed by several meta-analyses in Asian population [21–23]. Furthermore, a recent meta-analysis showed that the higher level of plasma total homocysteine was associated with the higher risk of SCZ [24]. Interestingly, the C677T polymorphism also played a critical role in the positive and negative symptoms of SCZ. For example, Roffman et al. found that SCZ patients with the TT genotype exhibited greater deficits in the verbal fluency test and more difficulties in the Wisconsin Card Sorting Test, but not in California Verbal Learning Test performance [25, 26]. In particular, a study in the Chinese population revealed that the TT genotype affected gray matter density and impaired memory in SCZ patients [27]. Taken together, these studies indicate that the C677T polymorphism may be involved in the psychopathology of SCZ patients.

Considering the significant sex differences in SCZ patients, the pathogenic role of *MTHFR* gene and cognitive impairment of SCZ, it is interesting to investigate whether there was a sex difference between the C667T polymorphism and cognitive impairment and clinical symptoms of SCZ patients. In particular, Wan et al. reported that male-specific effects of *MTHFR* polymorphism on the symptoms in chronic SZ patients [28]. However, the sample size is small and only one cognitive function domain was measured in that study. Based on previous literature, we hypothesized that when the patients were stratified by sex, the TT genotype of C677T was differently associated with symptoms and cognitive impairment in SCZ. Therefore, the purpose of the present study was to examine whether the interactive effects between C677T and sex would affect the clinical manifestations of SCZ patients.

## RESULTS

### Subject characteristics

The demographic and clinical characteristics were depicted in Table 1. There were significant differences in sex, age, smoking status and BMI between patients and healthy controls (all p< 0.01), which were controlled as confounding factors in the following analyses.

**Table 1:** Demographic characteristics, clinical data and *MTHFR* C677T genotypes in schizophrenia vs healthy controls.

**Variable**	**SCZ (n=957)**	**Controls (n=576)**	**F or χ^2^ (p)**
Sex (male/female)	783/174	263/313	216.8 (< 0.001)
Age (ys)	47.8 ± 10.2	45.8 ± 12.8	11.7 (<0 .001)
Education (ys)	9.3 ± 6.4	8.7 ± 3.2	1.5 (0.09)
Smokers/Nonsmokers	627/318	218/358	117.7(<0 .001)
BMI (kg/m^2^)	24.5 ± 3.9	25.1 ± 3.9	2.7 (<0 .01)
Drinking	727/137	455/121	6.2(0.013)
Age at onset, mean ± SD, ys	23.5 ± 5.7		
Duration of illness, mean ± SD, ys	24.3 ± 10.0		
Antipsychotic dose (mg/d)	455.2 ± 418.4		

Notes: *SCZ* schizophrenia; ys years *BMI* body mass index.

### Sex difference in the associations between C677T polymorphism and clinical symptoms in patients

The distribution of MTHFR C677T genotypes were in HWE in both patients and controls (both p>0.05). After adjusting for sex, smoking, body mass index (BMI) and age, there was no significant difference in the frequencies of C677T allele and genotype between SCZ patients and healthy controls (both p>0.05). After further analysis by sex grouping, there were significant differences in the frequencies of C677T genotype and allele between male patients and male controls (*χ^2^* =7.6, p=0.023; *χ^2^* =5.9, p=0.019, respectively, Table 2), but there was no significant difference between female patients and female controls (all p>0.05). Compared with the male control group, the prevalence of C677T-TT homozygous genotype was slightly higher in male patients (40.5% vs 28.9%). There was no significant difference in females (all p>0.05). Moreover, compared with C677T-CC heterozygous males, homozygous C677T-TT males were approximate 1.5-fold more likely to have SCZ (OR=1.51, 95% CI=1.0-2.3, p=0.042).

**Table 2:** *MTHFR* C677T allele and genotype frequencies in male and female of schizophrenia and healthy controls.

**Variable**	**SCZ (n=957)**	**Controls (n=576)**	**F or χ^2^ (p)**
C677T genotype			2.0(0.36)
CC (%)	298(38.9)	145(34.8)	
CT (%)	344(45.0)	202(48.4)	
TT (%)	123(16.1)	70(16.8)	
Male (CC/CT/TT)			7.6(0.023)
CC (%)	101(15.8)	31(18.7)	
CT (%)	280(43.7)	87(52.4)	
TT (%)	260(40.5)	48(28.9)	
Female (CC/CT/TT)			2.3(0.32)
CC (%)	22(17.7)	38(15.3)	
CT (%)	64(51.6)	115(46.2)	
TT (%)	38(30.6)	96(38.5)	
C677T allele (C/T)	590/940	342/492	0.08(0.83)
Male (C/T)	482/800	149/183	5.9(0.015)
Female (C/T)	108/140	191/307	1.9(0.17)

There were significant differences in symptoms between male and female patients. Male patients had less severe positive symptoms, negative symptoms, general symptoms and PANSS total score than female patients (all p<0.05).

**Figure 1:**
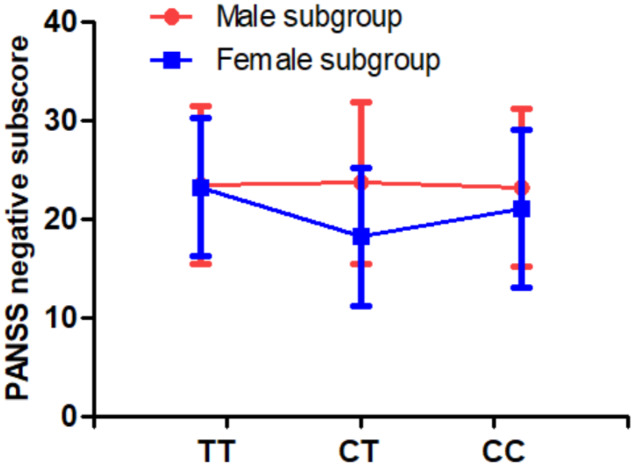
**There was a significant interaction of sex and *MTHFR C677T* genotype on PANSS negative symptoms (p=0.015).**

In addition, we found that there were differences in PANSS negative symptoms between *MTHFR* C677T genotypes (p=0.031). Multivariate analysis of covariance also revealed a significant sex difference in the association between C677T genotype and PANSS negative symptoms (p=0.015, Table 3 and Figure 1). Further analysis found that female patients with CT genotype had fewer severe negative symptoms than male patients with CT genotype.

**Table 3:** Sex differences of clinical symptoms and cognitive functions in the patients with schizophrenia and healthy controls.

	**Male**			**Female**			**Sex p**	**Genotype p**	**Sex×gene p**
	**TT**	**CT**	**CC**	**TT**	**CT**	**CC**			
Patients with SCZ									
**Cognitive functions**									
Immediate memory	56.3±14.5	58.0±15.7	58.4±16.8	64.0±13.9	68.8±22.9	58.7±13.9	0.017	0.06	0.05
Visuospatial/constructional	75.3±17.9	76.5±18.2	78.0±20.1	81.0±18.1	83.9±19.1	84.9±22.3	0.010	0.58	0.95
Language	81.0±14.6	80.7±15.4	80.3±16.6	83.3±16.3	88.9±15.8	80.7±15.0	0.084	0.23	0.24
Attention	67.5±17.3	70.0±16.7	71.1±18.6	83.0±15.2	78.3±17.1	71.5±17.8	0.001	0.46	0.05
Delayed memory	62.3±17.7	67.2±19.1	65.0±19.2	76.4±20.3	79.3±23.0	67.9±17.4	<0.001	0.04	0.26
Total score	61.7±13.4	63.5±14.6	64.4±14.8	71.8±14.4	75.0±19.0	65.9±14.9	<0.001	0.22	0.14
Clinical symptoms									
Positive symptom	11.2±4.5	11.1±4.6	11.5±4.4	13.2±5.6	14.9±6.6	14.0±6.7	<0.001	0.37	0.24
Negative symptom	23.5±8.0	23.7±8.2	23.2±8.0	23.2±7.3	18.2±9.0	21.0±8.1	0.002	0.031	0.015
General psychopathology	25.0±5.5	24.7±5.6	25.5±6.2	28.8±6.3	27.4±5.6	28.8±8.5	<0.001	0.27	0.72
PANSS total score	59.7±14.5	59.5±14.6	60.2±14.7	65.2±14.6	60.5±14.8	63.7±19.4	0.04	0.29	0.39
Healthy controls									
**Cognitive functions**									
Immediate memory	74.9±17.0	76.2±15.3	74.4±15.7	73.3±17.3	77.8±18.3	74.6±20.0	0.97	0.32	0.74
Visuospatial/constructional	80.4±15.1	82.1±16.6	80.3±14.7	77.1±14.9	79.7±16.9	77.4±12.2	0.11	0.44	0.97
Language	95.2±9.1	94.0±12.0	97.5±13.4	93.0±14.1	93.3±13.3	92.3±16.5	0.08	0.80	0.48
Attention	87.5±21.0	90.5±16.3	87.9±20.5	83.5±22.0	85.9±22.4	92.9±15.6	0.61	0.27	0.24
Delayed memory	89.2±12.5	84.7±14.4	88.7±15.5	85.0±16.1	87.1±16.3	85.6±14.5	0.31	0.79	0.12
Total score	80.6±13.6	81.1±13.1	81.9±15.2	77.5±15.9	80.2±16.6	80.0±15.1	0.26	0.60	0.83

### Sex difference in the associations between MTHFR C677T polymorphism and cognitive functions both in patients and controls

Female patients performed better than male patients in terms of delayed memory, attention, visuospatial/constructional, immediate memory and RBANS total score (all p<0.05). Moreover, we found that the **C677T genotype was related to delayed memory index (F=3.4, p=0.036, Table 3) in patients, but not in controls. There was no significant difference in cognitive function between patients using typical and atypical antipsychotics.

**Figure 2:**
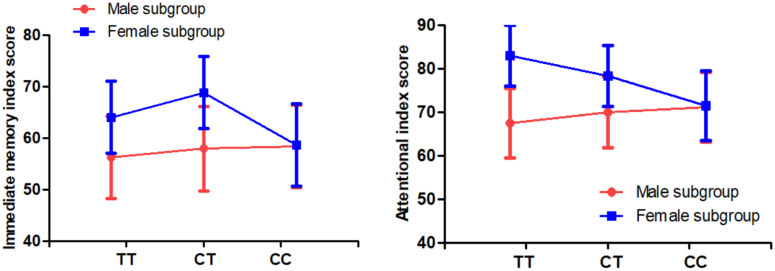
**There were significant interactions of sex and *MTHFR C677T* genotypes on immediate memory and attention index score in the patients with SCZ (all p<0.05).**

Multivariate analysis of covariance also revealed that there was a trend toward a significant sex difference in the relationship between C677T genotype and immediate memory and attention in patients (all p=0.05), but not in controls (all p>0.05). Further analysis found that female patients with TT and CT genotypes performed better on immediate memory and attention than male patients with TT and CT genotypes, while female patients with CC genotype had similar cognitive function as male patients with CC genotype (Table 3 and Figure 2).

## DISCUSSION

There were three major findings in the current study. First, the C677T polymorphism was susceptible to SCZ in male patients and was associated with PANSS negative symptoms and RBANS cognitive impairment. Second, except for RBANS language index, the RBANS total score and all 5 index scores in male patients were lower than those in female patients. Third, there were significant sex differences in the association between C677T polymorphism and negative symptoms and cognitive impairment (including immediate memory and attention), suggesting that the multifactorial etiology involving *MTHFR* gene and sex may be involved in the pathophysiology of SCZ patients.

Some previous studies have examined the possible impact of *MTHFR* gene on susceptibility to SCZ, but the results vary in different populations. Our findings provide more evidence that *MTHFR* C677T polymorphism is a risk factor for Chinese male SCZ patients. Then, we found that this polymorphism was correlated to delayed memory in SCZ patients, which was in accordance to previous studies [26, 29–31]. For example, Kontis et al. found that there was a significant interactive effect between *MTHFR* and *COMT* genes on spatial working memory in chronic SCZ patients and healthy controls [29]. Our results were comparable to those found by Kontis, supporting that the memory loss in SCZ patients was influenced by the *MTHFR* gene. However, we did not find a relationship between C677T and cognition in controls, which was inconsistent with the finding of Kontis et al. [29]. In this study, male patients performed worse than female patients on most cognitive tasks measured by RBANS, which was in line with most previous studies [32–34]. A possible explanation for the better cognitive function of female than male subjects may be associated with the influence of sex hormones on cognitive function. Studies have shown that testosterone and estrogen may play a critical role in cognition through the effects of dopamine and serotonin on certain brain regions, as reviewed in previous reports [35, 36]. In particular, the findings of preclinical studies demonstrated that estrogen can reduce the concentrations of dopamine and the number of dopamine receptors in the hippocampus [37–39]. Moreover, numerous studies have revealed that the average age of onset of symptoms in female SCZ patients was later than that in male patients [40, 41]. The neurodevelopmental hypothesis postulated that SCZ patients with an earlier onset age have more severe central nervous system damage and poorer cognitive function than those with later age of onset [14, 42, 43]. Therefore, estrogen may be the reason why female patients perform better in several domains of cognitive functions. Also, in this study, we found that men had more severe negative symptoms than women, which may be the reason for the decline in cognitive function. However, the exact mechanisms of male disadvantage in multiple cognitive domains warrants further preclinical and clinical investigations.

Another finding of this study was that an interplay effect of sex and C677T polymorphism on negative symptoms was found in patients. Female patients with CT genotype had fewer negative symptoms than female patients with CC and TT genotypes and male patients, suggesting that C677T genotype had heterozygous effect on negative symptom. Our results were partially consistent to previous studies in SCZ patients [25, 28]. Interestingly, we also found a relationship between C677T polymorphism, sex and cognitive function or negative symptoms in patients, but not in healthy controls. This is consistent with a previous study by Roffman et al. [26], which found that the *MTHFR* gene impaired cognitive function, taking into account the mediating effect of negative symptoms. We speculate that the interactive effect between CT genotype × sex and cognitive impairment may be associated with altered MTHFR enzyme activity. Studies have shown that *MTHFR* gene was correlated with MTHFR enzyme activity, and individuals with CT and TT genotypes have only 67% and 25% of enzyme activity of their CC genotype counterparts, respectively [17].

The lower enzyme activity in male patients may elevate the concentration of homocysteine in patients with insufficient dietary folate intake [44, 45]. A large number of studies have also demonstrated that elevated total homocysteine levels in blood were one of the most important risk factors for cognitive impairment in SCZ, and lowering the homocysteine concentration in patients with hyperhomocysteinemia may predict the improvement of cognitive function [46, 47]. However, it is worth mentioning that the reason why this C677T polymorphism was associated with negative symptoms and cognition may be due to the enzymatic effects of genotypes, which is only our speculation. According to previous studies on MTHFR enzyme activity, patients with homozygous wildtype CC should have fewer negative symptoms and better cognitive function than patients with homozygous (TT) and heterozygous (CT) genotypes. However, the fact is that the negative symptoms of patients with TT and CC genotype were almost equal, and the difference is not significantly (Table 3). In female patients, although there were significant differences in cognitive function between TT and CC genotype subjects, CC subjects were worse than TT subjects, which exceed our expectations. Taken together, this interpretation of the association between genotypes and negative symptoms or cognition due to the enzymatic effects of genotypes is quite speculative and may only provide part of the reason. The mechanism connecting *MTHFR* C677T gene polymorphism and SCZ is still unknown. In order to fully understand the relationship between *MTHFR* genetic variants and SCZ, it will be necessary to further combine multiple *MTHFR* genetic polymorphisms in larger samples for haplotype analysis.

There are several limitations that should be noted. First, the influence of *MTHFR* TT genotype on MTHFR enzyme activities was partially weakened by taking more folate and cobalamin [48]. However, in the present study, plasma folate and homocysteine levels of individuals were not measured. Also, we did not collect relevant information, nor did we know whether the participants recruited in this study were folic-acid-fortified people. Second, many studies have found that other factors may exacerbate folate deficiency, for example, older age, obesity and alcoholism [49, 50], which, unfortunately were not collected in this study. Third, there were more male than female subjects in SCZ patients, which may have led to bias in statistical analysis due to the imbalance of the number of subjects in each sex category in this study. Fourth, because atypical and typical antipsychotics have different effects on cognitive function, and patients were treated with different types of antipsychotics in this study, we could not exclude the effects of antipsychotic drugs.

In summary, in the pilot study, we showed the relationship between the *MTHFR* C677T polymorphism and SCZ in male patients, and this polymorphism was significantly associated with symptoms. Moreover, the C677T polymorphism was correlated with the delayed memory of the patients. Female patients had fewer symptoms and better cognition than male patients. We observed sex difference in the relationship between C677T polymorphism and negative symptoms, immediate memory and attention index scores. However, caution is needed in interpreting the results. Considering the small sample size and the different sex composition, the significant association between *MTHFR* C677T polymorphism and symptoms or cognition may be due to statistical bias. The sex difference in the relationship between MTHFR C677T polymorphism and clinical characteristics needs to be further studied in a larger sample of more female patients.

## MATERIALS AND METHODS

### Subjects

The participants were recruited from January 10, 2010 to December 25, 2013. A total of 957 SCZ patients were enrolled from Beijing Huilongguan Hospital and HeBei Province Veteran Psychiatric Hospital located in northern China. The inclusion criteria were as follows: (1) between the ages of 20 to 75 years; (2) Han Chinese; (3) diagnosed as SCZ confirmed by the Diagnostic and Statistical Manual of Mental Disorders, Fourth Edition (DSM-IV); (4) at least 60 months of illness; (5) taking a stable dose of antipsychotics for at least six months; (6) providing written informed consent. Antipsychotic drugs mainly consisted of drug monotherapy, including risperidone (n =210), clozapine (n= 430), sulpiride (n =49), chlorpromazine (n =69), quetiapine (n = 42), perphenazine (n = 45), haloperidol (n = 34), aripirazole (n = 29), and others (n = 49). The average daily dose of antipsychotics (in term of chlorpromazine equivalent) was 438 ± 407 mg/day.

During the same period, 576 healthy controls were randomly recruited through advertisements in the local community. Six psychiatrists screened and excluded potential controls with Axis I disorders at present or in their lifetime after a structured clinical interview. All control subjects were Han Chinese.

All subjects underwent extensive physical examinations and laboratory tests when they were recruited. Any participants with abnormal health were excluded. All subjects signed the written informed consent, which was approved by the Institutional Review Board of Beijing HuiLongGuan hospital.

### Assessments

The 4 clinical psychiatrists evaluated the psychiatric symptoms of SCZ by the Positive and Negative Syndrome Scale (PANSS) on the same day of blood sampling. In this study, these psychiatrists were trained to assess PANSS. After training, they maintained a correlation coefficient ≥0.8 for the reliability of the repeated evaluation of PANSS total score. The Repeatable Battery for the Assessment of Neuropsychological Status (RBANS, Form A) was used to assess the cognitive function of all participants. RBANS consists of five index scores and a total score. The five indexes include immediate memory, attention, visuospatial/constructional, language and delayed memory. Our group has translated RBANS into Chinese and established the clinical validity and test-retest reliability of the Chinese version among SCZ patients and controls.

### C677T polymorphism analysis

C677T polymorphism was genotyped by MALDI-TOF MS platform (Sequenom, CA, USA), following the standard procedure as described in sequenom genotyping protocol, which is considered efficient and accurate [51]. The MassARRAY system is an extensible platform that provides a set of applications for quantitative and qualitative genomic analysis. Primers for PCR and single base extension were designed by using the Sequenom MassARRAY Assay Designer software v3. The primers of C677T were: sense: 5′-ACGTTGGATGCTTGAAGGAGAAGGTGTCTG-3′, antisense: 5′- ACGTTGGATGCTTCACAAAGCGGAAGAATG-3′, and extent probe: AAGGTGTCTGCGGGAG.

The sample genotyping success rate in this study averaged 98.9%. 5% of all DNA samples were repeated, showing 99.5% reproducibility of SNP results.

### Statistical analysis

Hardy-Weinberg equilibrium (HWE) was examined using *χ^2^* test. The differences in the allele and genotype frequencies of C677T polymorphism between the two groups were analyzed by using *χ^2^* test. Then, analysis of variance (ANOVA) was performed to analyze the association between sex or *MTHFR* genotype and cognitive function or symptoms. Confounding factors included age, smoking, BMI, education, course of illness, antipsychotic type and dose. We paid more attention to the interaction term (sex × *MTHFR* genotype) because there may be any potential differences between different groups.

We used SPSS version 15.0 to perform all statistical analyses, with two-tailed p values of less than 0.05. Bonferroni correction was used for each analysis to correct multiple tests. The power of the sample was calculated by using Quanto software, with the relative risk and known risk allele frequencies under log additive, recessive and dominant models, respectively.
